# Updates on Anticoagulation and Laboratory Tools for Therapy Monitoring of Heparin, Vitamin K Antagonists and Direct Oral Anticoagulants

**DOI:** 10.3390/biomedicines9030264

**Published:** 2021-03-07

**Authors:** Osamu Kumano, Kohei Akatsuchi, Jean Amiral

**Affiliations:** 1 Research Department, HYPHEN BioMed, 155 Rue d’Eragny, 95000 Neuville sur Oise, France; jamiral@hyphen-biomed.com; 2 Protein Technology, Engineering 1, Sysmex Corporation, Kobe 651-2271, Japan; 3 R&D Division, Sysmex R&D Center Americas, Inc., Mundelein, IL 60060, USA; akatsuchik@Sysmex.com

**Keywords:** prothrombin time, activated partial thromboplastin time, heparin, warfarin, direct oral anticoagulants

## Abstract

Anticoagulant drugs have been used to prevent and treat thrombosis. However, they are associated with risk of hemorrhage. Therefore, prior to their clinical use, it is important to assess the risk of bleeding and thrombosis. In case of older anticoagulant drugs like heparin and warfarin, dose adjustment is required owing to narrow therapeutic ranges. The established monitoring methods for heparin and warfarin are activated partial thromboplastin time (APTT)/anti-Xa assay and prothrombin time – international normalized ratio (PT-INR), respectively. Since 2008, new generation anticoagulant drugs, called direct oral anticoagulants (DOACs), have been widely prescribed to prevent and treat several thromboembolic diseases. Although the use of DOACs without routine monitoring and frequent dose adjustment has been shown to be safe and effective, there may be clinical circumstances in specific patients when measurement of the anticoagulant effects of DOACs is required. Recently, anticoagulation therapy has received attention when treating patients with coronavirus disease 2019 (COVID-19). In this review, we discuss the mechanisms of anticoagulant drugs—heparin, warfarin, and DOACs and describe the methods used for the measurement of their effects. In addition, we discuss the latest findings on thrombosis mechanism in patients with COVID-19 with respect to biological chemistry.

## 1. Introduction

Thrombosis disorders require prompt treatment with anticoagulant drugs at therapeutic doses. Although these drugs are effective and useful for the prevention and treatment for thrombosis, the drugs are associated with the occurrence of hemorrhage, and therefore, the identification of patients at increased risk of bleeding is clinically important for selecting the optimal treatment and duration of anticoagulant therapy. Traditionally, heparin—consisting of unfractionated heparin (UFH), low molecular weight heparin (LMWH), and fondaparinux—is used widely. Heparin binds to antithrombin via its pentasaccharide, catalyzing the inactivation of thrombin and other clotting factors [1]. Warfarin, a vitamin K antagonist used to hamper γ-carboxylation of vitamin K-dependent coagulation factors, has also been used historically as an anticoagulant [1,2]. Drug monitoring in clinical laboratory tests is required when using these drugs. In recent years, direct oral anticoagulants (DOACs) have been developed, including direct factor IIa (i.e., dabigatran) and factor Xa inhibitors (i.e., rivaroxaban, apixaban, and edoxaban), which can overcome several of the limitations of warfarin treatment, such as food interactions and the need for frequent monitoring using clinical laboratory tests. DOACs are reported to have a superior safety profile compared with warfarin in some thrombosis disorders [3,4,5,6,7,8,9,10]. Nevertheless, even with the new generation of anticoagulant agents, the most relevant and frequent complication of anticoagulant treatment is major hemorrhage, which is associated with significant morbidity, mortality, and considerable costs [11,12,13]. Although routine monitoring of these drugs is not required, assessment of anticoagulant effect may be desirable in special situations and assessment of the individual bleeding risk might be relevant while considering the selection of the appropriate anticoagulant drug and treatment duration [14].

In addition to the introduction of new anticoagulant drugs, anticoagulation therapy has received attention with regard to treating patients suffering from coronavirus disease 2019 (COVID-19). Patients with COVID-19 associated pneumonia exhibit abnormal coagulation and organ dysfunction, and coagulopathy based on abnormal coagulation has been associated with a higher mortality rate [15,16]. Thus, a suitable treatment for the coagulopathy and thrombosis is required.

This review covers the anticoagulant mechanisms of heparin, warfarin, and DOACs as well as the measurement methods for these drugs. In addition, we attempt to provide the latest information on thrombosis mechanism in COVID-19 from the view of biological chemistry.

## 2. Anticoagulants

### 2.1. Heparin

#### 2.1.1. The Properties of Heparin

**Figure 1:**
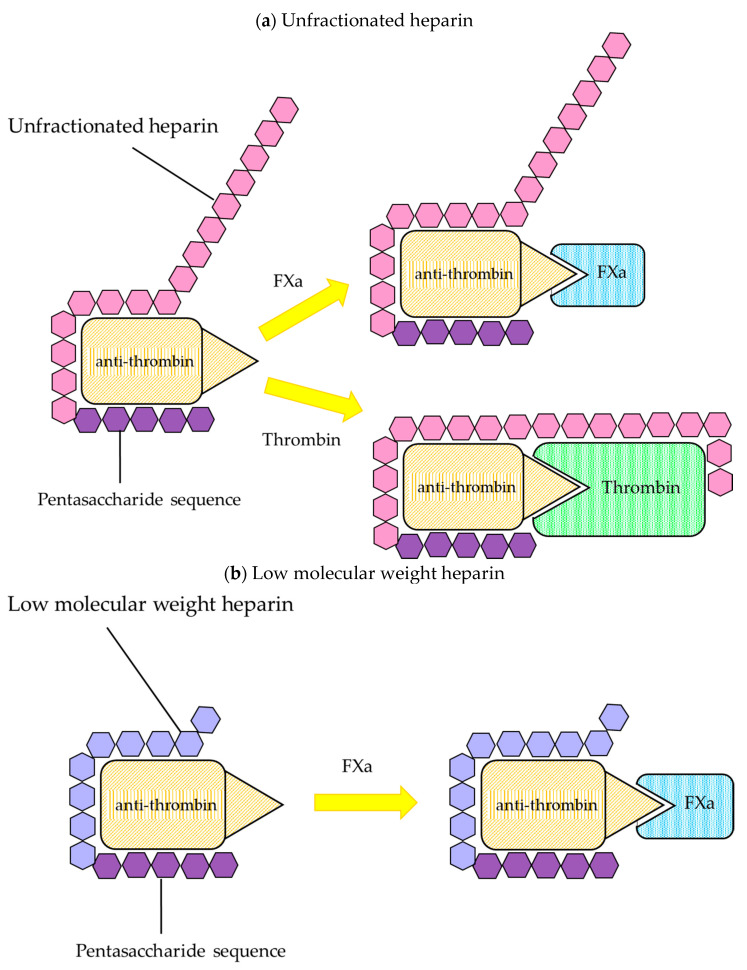
Inactivation of coagulation factors by heparin. Heparin binds to antithrombin (AT) through a high affinity pentasaccharide sequence. AT conformational structure is changed by binding to heparin, and it accelerates the interaction with thrombin or factor Xa. (**a**) Unfractionated heparin (UFH) can inactivate both thrombin and factor Xa. High molecular weight heparin is required to bind to thrombin. (**b**) Low molecular weight heparin (LMWH) catalyzes the inactivation of factor Xa by antithrombin. At least 18 saccharide units are required to catalyze the inactivation. LMWH has less inhibitory activity against thrombin than UFH.

Heparin is one of the oldest biological anticoagulant drugs and its antithrombotic properties were discovered by McLean almost 100 years ago [17]. Heparin is a heterogenous mixture of branched glycosaminoglycans and acts as an indirect anticoagulant, requiring antithrombin (AT). The main anticoagulant action of heparin is mediated by the heparin/AT interaction (Figure 1) [18]. In the heterogenous mixture of glycosaminoglycans, heparin contains a large number of sulfate groups which are negatively charged in the molecule. The negative charges bind to lysine sites on AT, producing a conformational change at the arginine reactive center, which converts AT from a slow, progressive thrombin inhibitor to a very rapid inhibitor. The arginine reactive center on the AT molecule binds covalently to the active center serine of thrombin and other coagulation enzymes, thereby irreversibly inhibiting their procoagulant activity [1,18].

Heparin has been widely used for the prevention and treatment of venous thrombosis, treatment of thromboembolism and disseminated intravascular coagulation (DIC), and prevention of clotting in artificial dialysis and extracorporeal circulation, et cetera. Two types of heparin are used clinically: UFH with a molecular weight of about 30,000–35,000 and LMWH with a molecular weight of about 4000–6000. The concept that the ability of heparin molecules to inactivate thrombin and other activated coagulation factors is chain length-dependent introduced the development of LMWH, which inhibits factor Xa mainly based on the presence of the high-affinity pentasaccharide [1,19,20,21].

#### 2.1.2. Biological Properties

##### Unfractionated Heparin

UFH is heterogenous with respect to molecular size, anticoagulant activity, and pharmacokinetic properties. Only about one third of an administered dose of UFH binds to AT, and this fraction is responsible for most of its anticoagulant effect [22,23]. The remaining two thirds of the dose has minimal anticoagulant activity at therapeutic concentrations. The heparin/AT complex inactivates thrombin and factors Xa, IXa, Xia, and XIIa. Thrombin and factor Xa are most sensitive to inhibition by heparin/AT, and thrombin is about 10-fold more sensitive to inhibition than factor Xa [1]. Although UFH binds to the coagulation factors and inactivates these enzymes, UFH also binds to several plasma proteins, endothelial cells, and macrophages, a property that further complicates its pharmacokinetics [24]. By inactivating thrombin, UFH not only prevents fibrin formation but also inhibits thrombin-induced activation of platelets and coagulation factors [1].

UFH is cleared, with a half-life of 60–90 min, in a nonlinear fashion through the combination of a rapid saturable mechanism and much slower first-order mechanisms [25,26,27]. One of the mechanisms reflects the binding of UFH to vascular endothelial cells, macrophages, and reticuloendothelial cells, resulting in a rapid and saturated elimination phase. UFH is depolymerized through this binding to cells [28]. The other mechanism is a slow elimination phase equivalent to renal clearance. Many endogenous plasma proteins, including histidine-rich glycoproteins, platelet factor 4, vitronectin, fibronectin, and von Willebrand factor bind to UFH, making it difficult to predict the pharmacokinetics of UFH [29].

##### Low Molecular Weight Heparin

LMWH may, in principle, be prepared by fractionation of UFH or by its depolymerization. It has an average molecular weight of 5000 Da. The affinity to endothelial cells is much lower than that of UFH, and LMWH does not bind to macrophages or reticuloendothelial cells, and its plasma half-life is 2–6 h, which is 2–4 times longer than that of UFH [30]. The molecules of heparin with <18 saccharides lose their ability to bind simultaneously to thrombin and AT, and therefore, are unable to catalyze thrombin inhibition [1]. In contrast, small heparin fragments, containing the high affinity pentasaccharide sequence, catalyze the inhibition of factor Xa by AT [31,32]. The synthetic pentasaccharide fondaparinux, unlike other products containing mixtures of fragments with different molecular weight, is a homogeneous product with a longer blood half-life, 17–21 h [29,33]. LMWH and fondaparinux have a very low affinity for heparin-binding plasma proteins and are largely removed by unsaturated renal filtration. Thus, LMWH lacks the nonspecific binding affinities of UFH, as a result, LMWH preparations have more predictable pharmacokinetic and pharmacodynamic properties.

##### 2.1.3. Monitoring of Heparin

Two different strategies are commonly used to monitor the therapeutic effects of UFH: the activated partial thromboplastin time (APTT) and the anti-Xa assay. The APTT is a global assay, which reflects both the intrinsic and common pathways of the coagulation cascade, and the clotting time is prolonged in the presence of heparin because heparin inhibits thrombin and factors Xa, IXa, XIa, XIIa, and VIIa. The anti-Xa assay is a chromogenic assay that measures inhibition of coagulation factor Xa. The assay is a kinetic method based on the inhibition of a constant amount, the reaction occurs in excess of factor Xa and the inhibition to factor Xa by heparin is assayed in the presence of AT. Hydrolysis of a chromogenic substrate specific for factor Xa by the residual factor Xa occurs, which cleaves the substrate. The amount of substrate released is inversely proportional to the concentration of heparin present.

A study conducted in 1972 established that the current standard of target value was 1.5–2.5 times greater than the upper limit of the institutional reference range in APTT reagent [34]. However, heparin sensitivity in APTT varies among the reagents and it was reported that appropriate targets ranged from 1.6 to 6.2 times the control value [1]. APTT reagents consist of activators that activate contact factors and phospholipids that serve as coagulation reaction scaffold for clotting factors. Commonly, ellagic acid and silica as activators and animals/plants-derived phospholipids or synthetic phospholipids are used. It was also reported that the sensitivity was largely dependent on the composition of phospholipids and phosphatidyl-serine concentration in total phospholipids used [35]. The APTT test has not been standardized for heparin therapy and target values may need to be adjusted by the manufacturer. The APTT values can be affected by various factors, including pre-analytic (sample collection and processing), analytic (reagent and instrument), and biologic factors (level of clotting factors). Especially, it could be difficult to collect blood for APTT heparin monitoring in an intensive care unit (ICU) because of the sample hematocrit, citrate anticoagulation concentration, high or low factor VIII, et cetera. Some guidelines recommend that each institution define its own therapeutic range by measuring an APTT value (corresponding to 0.3–0.7 IU/mL by factor Xa activity) used in the laboratory rather than a fixed APTT therapeutic range of 1.5–2.5 times the control value [1,36,37,38]. The anti-Xa assay is not affected by the above factors like APTT and has been proposed as a more accurate assay to find the concentration. The recommended anti-Xa therapeutic range is 0.3–0.7 IU/mL, and it is not necessary to establish the therapeutic range with each new lot of reagent because calibration curve would be created in each lot [39].

LMWH is typically administered in fixed doses, for thromboprophylaxis, or in total bodyweight-adjusted doses, for therapeutic effect [1]. Laboratory monitoring is generally not necessary. However, dose-finding trials have not been carried out in special population, such as patients with renal failure or severe obesity. It has been suggested that monitoring should be considered in such patients [40,41,42]. Although APTT is widely used for UFH, it is generally considered that APTT is unsuitable for LMWH monitoring because of its low sensitivity to LMWH [43]. While the anti-FXa assay is a reliable determinant of blood LMWH concentrations, it does not necessarily correlate well with the actual effect of the drug in vivo but represents the pharmacokinetics [44].

### 2.2. Warfarin

#### 2.2.1. Properties of Warfarin

**Figure 2:**
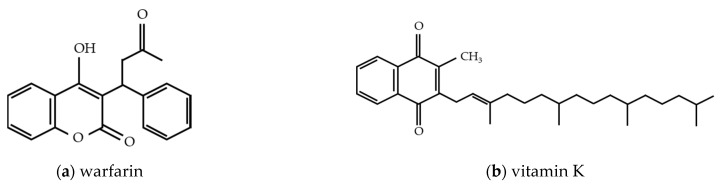
The chemical structure of (**a**) warfarin and vitamin (**a**) K.

Warfarin, an important anticoagulant used to prevent and treat thrombosis, was originally derived from dicoumarol, a coumarin that is primarily found in plants, such as sweet clover or melilot [45]. In 1939, the ingestion of sweet clover was identified as the cause of fatal hemorrhages in cattle. It was found by Campbell and Link (1944) that dicoumarol, a compound isolated from sweet clover, was the active hemorrhagic agent. Following this discovery, many synthetic anticoagulants were derived from coumarin [45,46,47]. Warfarin has an anticoagulant effect due to its structural resemblance to vitamin K (Figure 2). Vitamin K is an essential cofactor for γ-carboxylation of glutamate residues on coagulation factors II, VII, IX, and X, which are synthesized by the liver in their biologically inactive form. Carboxylation of these factors is directly related to the oxidation of vitamin K to its epoxide form. Formation of γ-carboxyglutamate residues results in a calcium-dependent conformational change that promotes the binding of cofactors to the phospholipid surface and results in coagulation. Warfarin can bind to vitamin K epoxide reductase, the enzyme that catalyzes the transformation of vitamin K epoxide back to vitamin K.

Warfarin is widely used as an oral anticoagulant. However, it has been reported that it interacts with many other drugs and also with foods that are rich in vitamin K and numerous factors can interfere with warfarin effects, such as the age, gender, bodyweight, liver and kidney function, genetic factors, adherence, and others [47,48]. Thus, the therapeutic range is narrow, and strict monitoring is required.

#### 2.2.2. Monitoring of Warfarin

Despite its therapeutic usefulness, the effectiveness of warfarin is largely dependent on the patient’s background and numerous other factors, and monitoring is required to keep the therapeutic range for the dose adjustment to control thrombosis and bleeding risk. For a long time, prothrombin time (PT) has been used as a monitoring parameter. PT represents the most commonly used coagulation test and is a single-stage screening test for extrinsic and common pathways. The reagent includes tissue factor, phospholipid, and calcium chloride; and this mixture is added to plasma, then fibrinogen is converted to fibrin. The test measures the time required for clot formation. The result, shown as the clotting time, is affected by the activity of coagulation factors II, V, VII, X, and fibrinogen, and the prolongation of PT clotting time may be due to one or more coagulation factor defects in extrinsic and common pathways, or indicate the presence of inhibitors against these factors, including drugs. Warfarin reduces the production of coagulation factor II, VII, IX, and X and the activity may also be decreased, and thus PT is elevated in warfarin patients and used as a parameter in monitoring tests.

Due to differing phospholipid compositions and sources of tissue factor, significant differences occur in the clotting time result from different PT reagents [49]. Initial efforts to standardize PT reagents was to establish the PT ratio (patient’s PT/mean normal PT). However, the standardization of PT ratio was unable to completely resolve the issue of reagents’ variability because the system could not eliminate discrepancies between the reagents [50]. For further standardization, international normalized ratio (INR) was developed, which reflects a mathematical calculation using PT ratio as further adjusted with a correction factor called international sensitivity index (ISI) [51].

INR = (Patient PT/Mean Normal PT) ^ISI^ (1)

**Figure 3:**
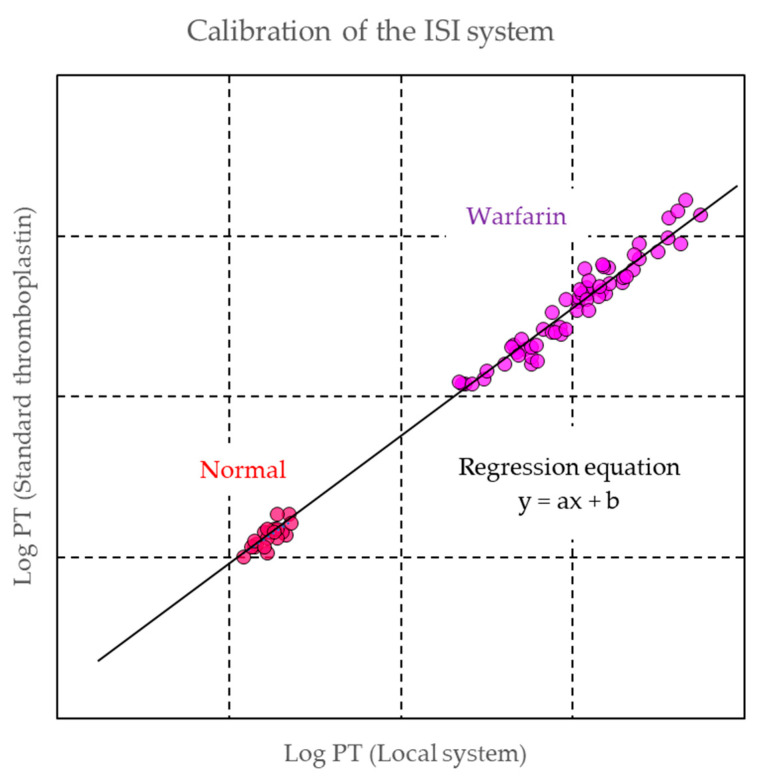
Example of a calibration line for determination of international sensitivity index (ISI). The prothrombin time (PT) clotting times of all samples including 20 normal (red) and 60 warfarin (purple) samples are measured by the employed instrument/reagent with the reference thromboplastin and the manual technique (WHO method), and the logarithms of the PT values employed are plotted against the logarithms of the PT values assigned by using WHO method. The regression equation is calculated, and the slope of the line is established. The ISI value of the employed instrument/reagent is calculated as: ISI = slope × ISI (ref) where ISI (ref) is the ISI of the reference thromboplastin.

Mean normal PT is calculated as the measurement value of PT clotting time in normal samples. ISI is a specific value assigned for each reagent by manufacturers. The ISI value is assigned by the method of The World Health Organization (WHO) Expert Committee on Biological Standardization with international reference preparations (IRP) of thromboplastins [52,53]. In this method, manually prepared PT with IRP is used for the tests, and commercial PT reagents from manufacturers are assessed and calibrated against the WHO method in 20 healthy subjects and 60 patients with stable conditions undergoing warfarin therapy. The values obtained from the manufactured PT reagent are plotted on the X-axis and the values from the WHO method on the Y-axis. The logarithm of these values is calculated, and the ISI value is obtained from the slope of the regression line in the correlation graph (Figure 3) [54]. For the verification of an established PT-INR system with the specific ISI value, it is recommended to measure 20–40 samples from stable patients with warfarin and 20 normal plasma samples [55].

Warfarin is effective for the primary and secondary prevention of venous thromboembolism (VTE), prevention of systemic embolism in patients with prosthetic heart valves or atrial fibrillation, prevention of stroke, recurrent infarction, and acute myocardial infarction [56,57,58,59]. However, bleeding is a major concern with warfarin therapy due to the influence of environmental factors and drug interactions in the setting of a narrow therapeutic range. Treatment with warfarin increases the risk of major bleeding by 0.3–0.5% per year and the risk of intracranial hemorrhage by approximately 0.2% per year compared with the controls. The most important risk factors for hemorrhage in warfarin therapy include intensity of anticoagulant effect, time within therapeutic range, and patient characteristics [60,61]. For these reasons, it is necessary to maintain the INR in the therapeutic range. A significant decrease in INR may increase the risk of thrombosis, mandating higher doses of warfarin. In contrast, a significant increase in INR represents an increased risk of bleeding, indicating a reduction in the dose or temporarily discontinuing the drug. Thus, accurate reporting of PT-INR results has a direct effect on the management of patients undergoing warfarin therapy. ISI has played an important role for the INR standardization to report the same INR value in different hospitals. This standardization system provides a historically optimized way to interpret PT results independently of the PT reagent used in institutions.

### 2.3. Direct Oral Anticoagulant (DOAC)

#### 2.3.1. Properties of DOAC

Since 2008, dabigatran, which is an oral direct thrombin inhibitor, and the direct factor Xa inhibitors (such as rivaroxaban, apixaban, and edoxaban) have been approved by the regulatory agencies. Subsequently, betrixaban, another anti-Xa direct oral anticoagulant, was approved for VTE prophylaxis in the United States. These anticoagulant drugs are widely prescribed to prevent and treat several kinds of thromboembolic diseases. Their pharmacokinetic and pharmacodynamic properties are more predictable than those of warfarin, and thus routine monitoring of the anticoagulant effect is not required [62]. During the start of the use of these drugs, the term—novel/new oral anticoagulants (NOACs)—was employed to refer to these drugs, especially, dabigatran, rivaroxaban, apixaban, and edoxaban because of the new class of oral anticoagulants and the term NOACs has been used for several years. However, they are not so new or novel anymore and the Subcommittee on the Control of Anticoagulation from Scientific and Standardization Committee of the International Society on Thrombosis and Haemostasis (ISTH-SSC) suggested using the term “direct oral anticoagulants” (DOACs) to reference the class of oral anticoagulants that directly inhibit a single target and have similar clinical properties [63].

**Figure 4:**
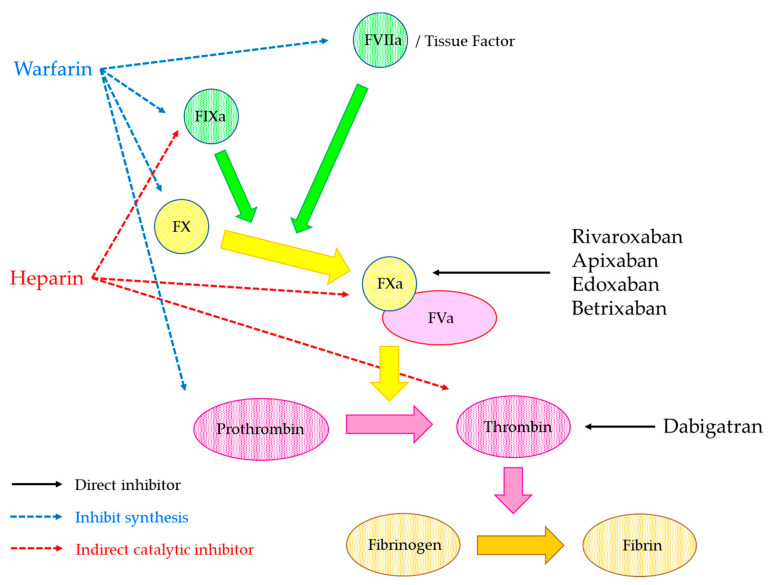
The inhibition mechanisms of direct oral anticoagulants (DOACs). Dabigatran is direct thrombin inhibitor, other DOACs including rivaroxaban, apixaban, edoxaban, and betrixaban are direct factor Xa inhibitors. DOACs inhibit coagulation factors directly. On the other hand, heparin indirectly inhibits coagulation factors through AT and warfarin affects to the coagulation factor synthesis.

DOACs directly inhibit coagulation factors, whilst warfarin inhibits the synthesis of coagulation factors (Figure 4). The characteristics of dabigatran, rivaroxaban, apixaban, and edoxaban are shown in Table 1. Dabigatran is generated from the oral prodrug, dabigatran etexilate, by the hydrolysis of esterases in the gut, liver, and blood [64]. Dabigatran which is the active form of dabigatran etexilate binds to the thrombin active site and inhibits free and fibrin-bond thrombin [65]. Rivaroxaban is a competitive inhibitor of FXa and it inhibits both free FXa and prothrombinase complex, and it reaches peak plasma concentration rapidly [66]. Apixaban is a direct and reversible inhibitor of FXa, and it inhibits free FXa, FXa in the prothrombinase complex, and FXa bound to platelets [64]. Edoxaban is also a highly selective, direct, and reversible inhibitor of FXa and it inhibits free FXa as well as that within the prothrombinase complex [67]. These drugs are rapidly becoming the most commonly prescribed oral anticoagulants for prevention of embolic stroke in patients with non-valvular atrial fibrillation (AF) and prevention and treatment of VTE [68]. Betrixaban is a new DOAC with a different pharmacokinetic profile than other DOACs. Betrixaban was recently approved for the indication of extended thromboprophylaxis in the United States based on the clinical trial compared with enoxaparin in hospitalized acute medically ill patients [69].

#### 2.3.2. Measurement of DOACs

The use of DOACs without routine monitoring and frequent dose adjustment has been shown to be safe and effective in the majority of patients, thus making them more convenient anticoagulants than warfarin [70]. However, it was reported that there would be clinical circumstances in specific patients when measurements for the anticoagulant effects of DOAC would be required. The following cases have been discussed for the utility of the measurements [62,71].

(i) Persistent bleeding or thrombosis
(ii) Decreased drug clearance resulting from impaired kidney function or liver disease
(iii) Identification of subtherapeutic or supratherapeutic levels in patients taking other drugs that are known to significantly affect pharmacokinetics
(iv) Extremely body weight: <40 kg or >120 kg
(v) Perioperative management
(vi) Reversal of anticoagulant
(vii) Suspicion of overdosage
(viii) Adherence to treatment protocol

The measurement assays have been developed to fulfill these requirements. It was considered that the optimal laboratory assays were dependent on the cases and the tests were classified as two types as follows: qualitative (presence or absence) and quantitative (ng/mL).

##### Qualitative Assays for DOACs

DOACs can interfere with clot-based coagulation assay because these drugs inhibit thrombin or factor Xa. Many researchers reported that the effect on clotting assays were largely dependent on principles like PT and APTT, which are the global assays for the screening of coagulation cascade. Endogenous coagulation activity changes and coagulation inhibitors can also affect the measurement results of PT and APTT, and these global assays lack the specificity for the measurements of DOACs anticoagulation. However, these tests have been performed as routine tests in many clinical laboratories, which indicates that the results are obtained in short turn-around-times. Timely evaluation is important because the half-lives of DOACs are short and it is critical in several scenarios, such as life-threatening bleeding or acute stroke management [72].

PT is prolonged by dabigatran and rivaroxaban in a concentration-dependent manner, and PT has higher sensitivity to rivaroxaban than dabigatran [73,74]. Specifically, the investigation for rivaroxaban has been performed by many researchers, it was reported that two-fold clotting times was 66–258 ng/mL among PT reagents in the spiked samples and it indicated that the variability among the reagents was wide. Although the relation was linear in the spiked samples, the correlation between PT and the concentration measured by mass spectrometry in clinical samples was weak (R^2^ = 0.3178) and the discrepancy samples were also recognized in the correlation [74,75]. The sensitivity to apixaban was lower than that of rivaroxaban. Although the sensitivity is largely dependent on PT reagents, PT may be normal with apixaban concentrations up to 200 ng/mL [76,77,78]. The sensitivity to edoxaban is equivalent or less than rivaroxaban, but PT is more sensitive than that of APTT, the prolongation of the PT is also concentration- and reagent-dependent [79,80]. Although INR and ISI are widely used from the view of standardization, this system had been established for warfarin monitoring based on VKA sensitivity. This system is not suitable for DOACs measurements and data interpretation [81].

It was also reported that APTT had sensitivity for dabigatran, rivaroxaban, apixaban, and edoxaban in concentration-dependent prolongation of clotting times. Particularly, the sensitivity to dabigatran was higher than those of other drugs [74,82,83] and the prolongation of APTT increased in a non-linear manner with increasing concentrations of dabigatran in clinical samples [84]. As described above, APTT is a commonly used parameter for the measurement of the degrees of anticoagulation in patients receiving anticoagulant medications. It has been used for the monitoring of treatment with UFH and has been discussed more recently in the clinical evaluation of direct thrombin inhibitors, such as hirudin [85,86,87]. Although commercial APTT reagents differ in their sensitivity, it was estimated that APTT had higher sensitivity to thrombin inhibitor than PT and the principle might be useful for the detection of dabigatran.

Overall, APTT is more sensitive with dabigatran and PT with rivaroxaban, whilst apixaban has very little influence on both PT and APTT. The specificity was low and the sensitivity to drugs is not so high. It indicates that the ability of these tests to quantify DOAC concentrations is poor and reagent-dependent. In addition, PT and APTT are not recommended at all for monitoring DOACs.

Thrombin time (TT) is highly sensitive to dabigatran because the reagent includes thrombin in lower concentration than fibrinogen reagent, converts fibrinogen into fibrin, and the clotting time is detected. Dabigatran concentrations in trough levels lower than 30 ng/mL lead to significant prolongation of the TT; a normal TT indicates that little or no dabigatran is present, but a prolonged TT does not necessarily equate to a high dabigatran level [88,89]. Thus, TT is only useful in the very low dabigatran concentration range to exclude its presence in plasma samples. The reagent is not affected by direct factor Xa inhibitor.

In addition to these classical coagulation tests, other assays had been developed to measure DOACs in clotting principles, and one of them is diluted prothrombin time (dPT). In routine PT tests, the quantity of thromboplastin is much higher than in physiological conditions. dPT experiments were performed to increase the sensitivity of the test because the conditions of this test might be closer to the actual physiological conditions. In these dPT experiments, PT reagents were diluted with 25 mM CaCl_2_ solution to obtain normal clotting times of 30–40 s. The dilution ratios were different in each reagent, but these were 1/8–1/750 (one part of reagent/x parts of CaCl_2_ solution) [73,90]. Although the results obtained with several dPT reagents still varied, depending on the reagents used, it was confirmed that the clotting times of dPT were prolonged in rivaroxaban samples and dPT assay demonstrated weak correlation with rivaroxaban concentrations. The sensitivity to rivaroxaban in dPT was increased as the dilution ratios became higher [75,90]. Ieko et al. developed a novel dPT assay with a new formula to normalize the effect of thrombin generation. They used 500-fold diluted PT reagent for the measurements, the results were expressed as the ratio of thrombin generation in their formula, and they investigated the correlation between the formula and the drug concentration in the samples of rivaroxaban, apixaban, and edoxaban. The results showed that the formula was positively correlated with the concentrations of all drugs, and it was also recognized that the formula showed a significant decrease in cases with thrombosis and increase in those with hemorrhage [91]. Recently, we also developed a modified dPT (mdPT) assay and reported that the reagent had good correlation against the drug concentration of rivaroxaban, apixaban, and edoxaban, and the sensitivity of mdPT was higher than that of commercial PT reagents in these clinical samples. In addition, the discrepancy analysis in the correlation between mdPT and the drug concentrations were performed, and the results showed that the discrepancy might be related to the lower coagulation activity of factors II, V, VII, and X. Thus, it was considered that mdPT reagent reflected not only DOAC inhibition but also coagulation factor activity and this assay may predict bleeding risk in patients treated with DOAC at a lower level of coagulation activity [92,93]. Letertre et al. developed an interesting assay based on dPT. They hypothesized that the levels of anticoagulant drugs could be measured with use of the dPT and diluted factor II and X deficient plasma-PT (dFiix-PT), which is only affected by reduced levels of active factor II and X. In the dFiix-PT assays, FII- and FX-depleted plasma was mixed with the test plasma to correct for any factor deficiency other than FII or FX, and the test plasma was mixed with diluted thromboplastin, CaCl_2_ solution, and factor II and X depleted plasma; and the clotting time was detected. This assay showed good correlation against drug concentration in dabigatran, rivaroxaban, and apixaban. They also showed that the dFiix-PT with drug-specific calibration curves estimated the drug concentration in plasma samples from patients taking these agents and suggested that the assay may be suitable for screening for the presence of anticoagulant drug in plasma and the test might be suitable for emergency testing of anticoagulant concentrations or effects [94]. Overall, while these global assays are not specific for DOAC, they are useful for screening for anticoagulant drugs and finding the effects of drug concentration with coagulation factor activity. Although further studies are required from the perspective of clinical insight, it is considered that these assays are helpful to understand the status of patients taking anticoagulants.

##### Quantitative Assay for DOAC Measurement

Liquid chromatography-mass spectrometry/mass spectrometry (LC-MS/MS) is considered the gold standard method for the quantitative assay for DOAC measurement because it has high degree of specificity, sensitivity, selectivity, and reproducibility, and this method is often used in clinical development to evaluate DOAC pharmacokinetics [95,96,97]. The lower limits of detection (LoD) and quantitation (LoQ) in LC-MS/MS for DOAC detection has been reported as 0.025–3 ng/mL and the reportable range of quantitation has also been reported as 5–500 ng/mL [98,99,100,101,102,103]. Although LC-MS/MS is considered the most accurate method for DOAC measurements, it is not widely used because of the limitation of availability and complexity associated with this testing. In addition, LC-MS/MS has a high intra- and inter-assay coefficient of variation and it needs to be calibrated with the purified drug, which contributes to series-to-series variations. Thus, the drug-calibrated clot-based or chromogenic methods have been developed and adapted to automated coagulation analyzers.

One of the clot-based kits for dabigatran measurement is Hemoclot thrombin inhibitor (Hyphen BioMed, Neuville-sur-Oise, France). For detection, the diluted tested plasma is mixed with normal pooled plasma. Then, clotting is initiated by addition of human thrombin and the clotting time is measured in coagulation analyzers. The clotting time is directly related to the concentration of dabigatran, and the concentration is calculated from the calibration curve obtained from the dabigatran calibrator. It was shown that the clotting method and LC-MS/MS analysis correlated well, and the agreement was good [98]. Stangier et al. also reported that this kit enabled the accurate assessment of dabigatran concentrations within the therapeutic concentration range without inter-laboratory variability [104]. The correlation between the clotting method and qualitative tests as APTT and thrombin time has also been investigated. The APTT demonstrated a modest correlation with the dabigatran concentration measured by the clotting method but the correlation became less reliable at higher dabigatran levels and it was recognized that the sensitivity was different among APTT reagents. The TT was sensitive to the presence of dabigatran and showed >300 s in more than 60 ng/mL concentration. It was confirmed that the TT was too sensitive to quantify dabigatran levels [105]. The other study also demonstrated that APTT and TT could not identify the drug presence in too low or too high concentrations, respectively [106]. For dabigatran concentration detection, it is considered that the clotting method is a more suitable assay. In addition, there was an accurate correlation reported for ecarin clotting time or ecarin chromogenic assay and dabigatran concentrations [74]. Although the upper limit of the measurements is dependent on the ecarin unit and the composition, it was reported that these assays could detect dabigatran up to 940 ng/mL.

Chromogenic anti-Xa assay has been used in clinical laboratories for several decades as a method to assess heparin concentration. This assay is appropriate for measuring direct factor Xa inhibitor drug concentration. The chromogenic anti-Xa assay principle is a kinetics method based on the inhibition of factor Xa at a constant and limiting concentration by the tested factor Xa inhibitor. The samples are mixed with an excess amount of factor Xa, and factor Xa inhibitor binds to factor Xa. The remaining factor Xa is then measured by its amidolytic activity on a factor Xa specific chromogenic substrate, and the residual factor Xa cleaves the substrate. The amount of cleaved substrate is inversely proportional to the concentration of factor Xa inhibitor in the tested samples. The concentration is calculated from the calibration curve obtained from rivaroxaban, apixaban, and edoxaban calibrator, respectively. It has been proved that this kit has the correlation against LC-MS/MS, and it was also shown that the inter variability was small [100,107,108]. Douxfils et al. reported that the correlation between PT and LC-MS/MS was not linear and the sensitivity to drugs differed among reagents [100], it could be difficult to perform the standardization to estimate drug concentration in PT reagents. Biophen DiXaI is useful to quantify drug concentration because the kit has high sensitivity to drugs and adapted to automated coagulation analyzers. DOACs have short half-lives, their concentration is time-dependent, and the concentration of peak time is quite different from that of trough time. The information when patients take the drugs is useful for the interpretation of the drug concentration data.

## 3. Coronavirus Disease 2019 and Thrombosis

### 3.1. The Thrombosis Mechanisms

**Figure 5:**
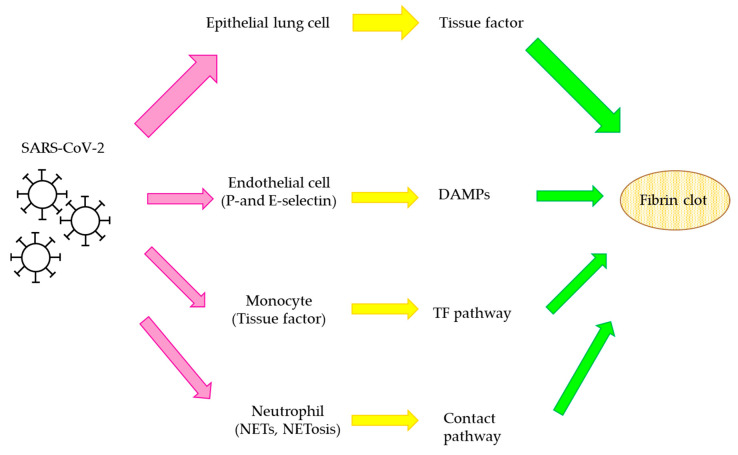
Proposed mechanisms of SARS-CoV-2 associated thrombosis. Several thrombosis mechanisms have been proposed, the important pathogenic effect is the binding and destruction of epithelial lung cells. The phenomenon relates to tissue factor release into blood circulation and activate coagulation pathways at lung sites and extend it throughout the circulatory systems. In addition, infected cells become the trigger to release proinflammatory cytokines and chemokines. The activated endothelial cells upregulate P-selectin and E-selectin leading to recruitment of platelets and leukocytes and complement activation. The infection and recruitment are related to the release of danger-associated molecular patterns (DAMPs). Monocytes activated through complement activation, express tissue factor, and activate coagulation cascades. Neutrophils release NETs which activate the contact pathway directly. Thrombin is generated through these pathways, fibrinogen is converted to fibrin, and the activation of coagulation cascade is related to SARS-CoV-2 associated thrombosis. The reaction of (i) affects to cells, (ii) protein expression, and (iii) the mechanisms of fibrin clot generation were shown as red, yellow and green arrows, respectively.

Anticoagulant drugs are effective for the primary and secondary prevention of VTE, prevention of systemic embolism, atrial fibrillation, et cetera [56,109]. In December 2019, a new viral infection occurred in Wuhan City, Hubei Province, China. Severe acute respiratory syndrome coronavirus 2 (SARS-CoV-2), a novel beta-coronavirus that can spread from human to human, is recognized as the causative virus of this infection. SARS-CoV-2 infects vascular endothelial cells through angiotensin-converting enzyme 2 (ACE2), the receptor for angiotensin II. The incubation period for SARS-CoV-2 is generally 5–6 d and once symptoms occur, the peak viremia occurs within 2–5 d of onset [110]. The virus was named COVID-19 and this infection is a mild disease in approximately 80% of patients and these patients usually present with symptoms of fever and a dry cough and it resolves spontaneously within 10 days. However, in almost 20% of patients, viral infection progresses down the trachea to the lungs [111]. The adverse effects of COVID-19 were initially thought to affect the primarily tract by causing pneumonia and acute respiratory distress syndrome (ARDS), but it is now known that COVID-19 is associated with the thrombosis promoting state which can cause microvascular thrombosis, venous, or arterial thrombosis [112]. Serious SARS-CoV-2 infection was often associated with coagulopathy, with increased D-dimer, which was more pronounced in critically ill patients [113]. The thrombosis mechanisms in COVID-19 patients are under current investigation and the most appropriate treatment is being discussed, with guidelines being issued by scientific societies. In addition, increased C-reactive protein (CRP) and interleukin-6 (IL-6) correlated with severity, suggesting an association between innate immune response and thrombotic response [114]. Host hypercytokinemia and massive proinflammatory responses may contribute to endothelial dysfunction of COVID-19, particularly in critically ill patients. Proinflammatory cells, cytokines, and chemokines not only activate the coagulation system, but also attenuate the anticoagulant mechanism and amplify the vicious circle of vascular coagulation and thrombosis [115]. In lung autopsies from COVID-19 patients, neutrophil pulmonary infiltration and neutrophil extracellular traps (NETs) formation were revealed and NET markers were reported to be elevated in critically ill hospitalized patients [116,117]. The NETs are the extracellular web of chromatin, bactericidal proteins, and oxidative enzymes released by neutrophils to contain infection. Thus, NET phenomenon could be related to the thrombosis in COVID-19 patients and NETs contribute to inflammation and microvascular thrombosis [118,119]. These complicated situations, including endothelial inflammation, complement activation, thrombin generation, and platelet/leukocyte thrombotic complications, are related to the thrombosis mechanisms in COVID-19 patients, the current proposed mechanisms of SARS-CoV-2 associated thrombosis are shown in Figure 5 [112].

### 3.2. COVID-19 Therapy and Anticoagulants

With the suspected contribution of thrombotic events to morbidity and mortality in COVID-19 patients, novel options for preventing COVID-19-related thrombotic diseases are being explored. However, the data is limited to determination of antithrombotic therapy to improve outcomes in patients with COVID-19 for whom evidence of thrombosis has not been confirmed. An interim guideline for the management of coagulopathy in COVID-19 patients was introduced by the International Society for Thrombosis and Hemostasis (ISTH) in March 2020. The guidelines recommend prophylactic administration of LMWH in all patients with COVID-19 who require hospitalization unless there is active bleeding or a contraindication, such as a platelet count <25 × 10^9^ /L [120]. It is also recommended by the WHO to use pharmacological prophylaxis in patients (adults and adolescents) hospitalized with COVID-19, such as low molecular weight heparin (e.g., enoxaparin) to prevent venous thromboembolism, when not contraindicated [121]. In addition, anticoagulant therapy using unfractionated heparin or low-molecular-weight heparin is recommended for reducing the depletion of coagulation substrates in patients with severe coagulation dysfunction when either fibrin/fibrinogen degradation products (FDP), D-dimer, or both, show either ≥10 μg/mL, ≥5 μg/mL, or both, respectively, in Chinese expert consensus [122]. For FDP reagents, it was reported that the reactivity to the fragments was different among the reagents and it should be considered for the data interpretation [123].

It has been reported that VTE frequency in subjects and a high incidence of pulmonary embolism admitted to the ICU for COVID-19 infection is increasing despite the use of standard prophylaxis for VTE with LMWH. Klok et al. reported that the occurrence of VTE was 27% and that of arterial vascular thrombosis was 3.7% despite the standard VTE prophylaxis with LMWH [124]. This was clearly higher than the 7.7% failure rate generally registered for VTE prophylaxis in ICUs [125]. White et al. demonstrated that heparin target concentrations were not reached, as measured by APTT or anti-Xa assays, in COVID-19 patients in ICU. Patient plasma spikes due to LMWH resulted in lower-than-expected anti-Xa levels in plasma. Based on these findings, the authors concluded that patients with COVID-19 were heparin resistant [126]. Decreased AT activity and increased levels of factor VIII and fibrinogen are cited as common predictors of heparin resistance [127]. Those COVID-19 patients have grossly raised fibrinogen and factor VIII levels with mild decreases in the AT [127], and these factors affect the APTT clotting times due to its global assay principle. Although there are bias risks in testing heparin in COVID-19 patients and the detailed thrombosis mechanisms are under investigation, anti-Xa assay might be more suitable for heparin monitoring of anticoagulant activity than APTT in some COVID-19 patient cases.

## 4. Conclusions

In this review, we described anticoagulant drugs, monitoring methods, thrombosis mechanisms, and therapy in COVID-19 patients. For the older anticoagulant drugs like heparin and warfarin, the monitoring methods have been established as APTT/anti-Xa assay and PT-INR, respectively. Although the therapeutic range is narrow and strict control is required, the sensitivity to these drugs is different among APTT/PT reagents and it may be difficult to control within the therapeutic range in some cases. To proceed with treatment with a suitable anticoagulant control, the clinical laboratory data should be interpreted carefully. Although the use of DOACs without routine monitoring and frequent dose adjustment has been shown to be safe and effective, there could be clinical circumstances in specific patients when measurements for the anticoagulant effects of DOACs would be required. Several assays have been developed and it is considered that the clinical laboratory tests might be useful to understand the patient status in some causes.

COVID-19 infection is related to thrombosis and coagulopathy. The data to understand the mechanisms and show the decision-making direction is limited. Further studies are desired for the better prediction and treatment.

## Figures and Tables

**Table 1:** The characteristics of DOACs.

	Dabigatran	Rivaroxaban	Apixaban	Edoxaban	Betrixaban
Target	Thrombin	Factor Xa	Factor Xa	Factor Xa	Factor Xa
Primary clearance	Renal	Renal	Fecal	Renal	Fecal
Tmax	1.5–3 h	2–3 h	3–4 h	1–2 h	3–4 h
Half-life	12–14 h	5–13 h	12 h	10–14 h	19–27 h
